# Investigating Dynamical Complexity and Fractal Characteristics of Bitcoin/US Dollar and Euro/US Dollar Exchange Rates around the COVID-19 Outbreak

**DOI:** 10.3390/e25020214

**Published:** 2023-01-22

**Authors:** Pavlos I. Zitis, Shinji Kakinaka, Ken Umeno, Michael P. Hanias, Stavros G. Stavrinides, Stelios M. Potirakis

**Affiliations:** 1 Department of Electrical and Electronics Engineering, University of West Attica, Ancient Olive Grove Campus, GR-12241 Aigaleo, Greece; 2 Department of Applied Mathematics and Physics, Graduate School of Informatics, Kyoto University, Sakyo, Kyoto 606-8501, Japan; 3 Department of Physics, International Hellenic University, GR-65404 Kavala, Greece; 4 Institute for Astronomy, Astrophysics, Space Applications and Remote Sensing, National Observatory of Athens, Metaxa and Vasileos Pavlou, GR-15236 Penteli, Greece

**Keywords:** COVID-19, bitcoin, cryptocurrencies, forex market, complexity, entropy, multifractal analysis, complex systems, financial crisis, econophysics

## Abstract

This article investigates the dynamical complexity and fractal characteristics changes of the Bitcoin/US dollar (BTC/USD) and Euro/US dollar (EUR/USD) returns in the period before and after the outbreak of the COVID-19 pandemic. More specifically, we applied the asymmetric multifractal detrended fluctuation analysis (A-MF-DFA) method to investigate the temporal evolution of the asymmetric multifractal spectrum parameters. In addition, we examined the temporal evolution of Fuzzy entropy, non-extensive Tsallis entropy, Shannon entropy, and Fisher information. Our research was motivated to contribute to the comprehension of the pandemic’s impact and the possible changes it caused in two currencies that play a key role in the modern financial system. Our results revealed that for the overall trend both before and after the outbreak of the pandemic, the BTC/USD returns exhibited persistent behavior while the EUR/USD returns exhibited anti-persistent behavior. Additionally, after the outbreak of COVID-19, there was an increase in the degree of multifractality, a dominance of large fluctuations, as well as a sharp decrease of the complexity (i.e., increase of the order and information content and decrease of randomness) of both BTC/USD and EUR/USD returns. The World Health Organization (WHO) announcement, in which COVID-19 was declared a global pandemic, appears to have had a significant impact on the sudden change in complexity. Our findings can help both investors and risk managers, as well as policymakers, to formulate a comprehensive response to the occurrence of such external events.

## 1. Introduction

Financial markets are widely recognized as typical examples of complex dynamical systems [1]. Asset prices are created by a large number of nonlinear interactions between heterogeneous agents and complex events occurring in the external environment [2,3]. The properties observed in financial time series such as nonlinearity, long-range dependence [4,5], volatility clustering [6], fat tails [7,8], asymmetry [9], chaos [10,11], fractals and multifractals [12,13], and self-similarity [14] have attracted the interest of many scientists from different fields. In the last three decades, physicists have studied and developed models to understand the behaviors and interactions in financial systems, establishing an interdisciplinary research field known as Econophysics [15,16,17]. This term first appeared in the published article by Stanley et al. [18] when analyzing the Dow Jones index; they found that stock returns followed a power law distribution. Since then, significant progress has been made in the field of Econophysics [19].

The dynamics of financial markets are difficult to understand not only because of the complexity of their internal elements but also because of the many intractable external factors acting on them. A recent example of an external factor causing disruptions in global financial markets is the outbreak of the COVID-19 pandemic. At its roots, the COVID-19 crisis is not a financial or economic crisis, it is a health crisis. Nevertheless, through its effects on supply and demand conditions, it evolved rapidly to a large-scale financial and economic crisis. In March 2020, the US stock market hit the circuit breaker mechanism four times in a period of ten days. Since its inception in 1987, the breaker has only ever been triggered once, in 1997. At the same time as the US crash, stock markets in Asia and Europe plunged also. More specifically, Japan’s stock market fell by more than 20%, while the UK’s main index, FTSE, fell by about 10.87% on 12 March 2020. Additionally, during the pandemic period, most economies experienced exchange rate volatility and currency depreciation due to capital outflows and market sentiments. Typical examples are the Australian dollar hitting a 17-year low of AUD 0.59215 and the New Zealand dollar hitting an 11-year low of NZD 0.5850. Furthermore, the price of gold dropped about 3.53%. It is worth noting that although gold is considered a strong safe haven for most developed markets during financial crises, there are findings showing that during the pandemic it was a weak safe haven for investors in the stock market [20]. The impact of COVID-19 affected even the newer asset classes such as cryptocurrencies. The declines in value of the three leading cryptocurrencies (Bitcoin, Ethereum, and Litecoin) exceeded 50% during the pandemic period.

The exchange rate is crucial for maintaining an economy’s external stability. As exchange rate directly associates with foreign debt, capital flows, trade balance, and export competitiveness, maintaining a stable exchange rate is one of the policymakers’ major concerns. On the other hand, several researchers argue that specific characteristics of cryptocurrencies, including the independence from monetary policy and the non-correlation with traditional assets, increase their resilience during crisis periods such as the recent pandemic crisis [21,22,23]. However, there is also the opposing view which argues that monetary policy has a significant impact on the price of cryptocurrencies as well as that the cryptocurrencies do not have zero correlation with other asset classes. For example, Chaoqun Ma et al. [24] found a strong response of Bitcoin prices to unexpected monetary policy actions, while Khanh Quoc Nguyen [25] found that S&P 500 returns significantly affected Bitcoin returns during the pandemic period. Therefore, it is concluded that understanding the pandemic’s impact and the possible changes it caused in the cryptocurrency and foreign exchange markets is crucial for both investors and risk managers as well as policymakers.

Particularly useful conclusions about the effects of COVID-19 on financial markets can be obtained by studying changes in the multifractality and complexity of financial time series during the period around the COVID-19 outbreak. In the field of Econophysics, extensive research has been conducted on these topics. For example, Mnif et al. [26] utilized the multifractal detrended fluctuation analysis (MF-DFA) approach to investigate the degree of cryptocurrency efficiency before and after the COVID-19 outbreak using a limited time period, until 19 May 2020. Their results indicated that the pandemic outbreak positively affected the efficiency of the five cryptocurrencies that they studied. Naeem et al. [9] examined the asymmetric efficiency of the cryptocurrencies Bitcoin, Ethereum, Litecoin, and Ripple, using 1-h data. In their analysis, the authors utilized the A-MF-DFA and their results showed that the price of cryptocurrencies exhibited significant asymmetric multifractality. Moreover, they found that uptrends showed stronger multifractality than downtrends. Additionally, applying the time-varying deficiency measure, they found that the pandemic outbreak had a negative impact on the efficiency of the cryptocurrencies that they analyzed. Kakinaka and Umeno [27], applying the A-MF-DFA, examined the asymmetric multifractality along with the market efficiency of two main cryptocurrencies (Bitcoin, Ethereum) during the pandemic period, taking into consideration different investment horizons. Their empirical results showed that the outbreak of COVID-19 affected the efficiency of the two cryptocurrencies differently in the short- and long-term horizons. More specifically, after the outbreak of COVID-19, Bitcoin and Ethereum in the short term exhibited stronger multifractality, while in the long term exhibited weaker multifractality. In addition, they studied the asymmetric market patterns between small and large price fluctuations and between upward and downward trends. These results confirmed that the outbreak caused a significant change in the level of asymmetry in cryptocurrency markets. Aslam et al. [28] applied the MF-DFA to study the efficiency of foreign exchange markets during the initial period of the COVID-19 pandemic. In their analysis, they used high-frequency data of six major currencies, during the period from 1 October 2019 to 31 March 2020. Before calculating the MF-DFA, they examined the inner dynamics of multifractality through seasonal and trend decomposition using loess. Their results indicated that efficiency of foreign exchange markets during the COVID-19 outbreak declined. Mensi et al. [29] examined the effect caused by the COVID-19 crisis on the pricing efficiency and asymmetric multifractality of major asset classes (US Treasury bond, US dollar index, S&P500, Brent oil, Gold, and Bitcoin). In their article, they applied the permutation entropy on intraday data from 30 April 2019 to 13 May 2020. Their results indicated that after the outbreak of COVID-19, the efficiency of all asset classes that they studied was deteriorated, and in most cases this deterioration was significant. In addition, using the A-MF-DFA, they found evidence of asymmetric multifractality in all markets. Drożdż et al. [30] studied the complexity of the cryptocurrency market in the period around the COVID-19 outbreak from three different perspectives. Their findings showed that throughout the time period analyzed, the returns of exchange rates were multifractal with intermittent signatures of bifractality that can be associated with the periods where the market was more volatile.

Lahmiri and Bekiros [31] investigated the time-varying characteristics of the informational efficiency in sixteen international stock markets and forty-five cryptocurrency markets before and during the pandemic period using the approximate entropy and Largest Lyapunov Exponent. Their results indicated that cryptocurrencies exhibited more irregularity and more instability during the pandemic period compared to international stock markets. Additionally, Lahmiri and Bekiros [32], applying Rényi entropy, analyzed the multiscale entropy function in the return time series of S&P500, Brent, WTI, Gas, Silver, Gold, Bitcoin, and VIX. Additionally, they analyzed the information sharing between these markets by estimating mutual information. Their results from Rényi entropy showed that for all market indices, disorder and randomness were more concentrated in less probable events. In addition, their results from the mutual information indicated that the information sharing network between markets has changed during the pandemic period. Wang J. and Wang X. [33] investigated the market efficiency of the S&P 500 Index, Gold, Bitcoin, and US Dollar Index during the extreme event of the COVID-19 pandemic using a multiscale entropy-based method. Their results indicated that, at all scales, the four markets’ efficiency decreased abruptly and persistently during the period from February to March 2020. Market efficiency decreased the most in the S&P 500 Index and the least in the Bitcoin market. Additionally, their results showed that Bitcoin market efficiency was more resilient than the others during the extreme event. Fernandes et al. [34] investigated the informational efficiency and price disorder of five main cryptocurrencies (Ethereum, Bitcoin, Cardano, XRP, and BNB) before and during the pandemic period. In their article, the authors applied the permutation entropy and Fisher information measure to construct the Shannon–Fisher causality plane in order to map the cryptocurrencies and their respective locations in a two-dimensional plane. Their results indicated that all cryptocurrencies exhibited high but slightly varying informational efficiency during both periods. Additionally, their results showed that Cardano was the most efficient cryptocurrency. Kim and Lee [35] investigated the evolution of the complexity of the cryptocurrency market and analyzed the properties from the previous upward trend market in 2017 against the COVID-19 pandemic. In their article, the authors used three popular measures of complexity based on the nonlinear analysis: sample entropy, approximate entropy, and Lempel–Ziv complexity. They studied the market complexity/unpredictability for forty-three cryptocurrency prices. They found that sample entropy, approximate entropy, and Lempel–Ziv complexity metrics of all markets could not generalize the COVID-19 effect of the complexity due to different patterns. Nevertheless, market unpredictability increased by the ongoing health crisis. Olbryś and Majewska [36] applied sample entropy to evaluate changes in the regularity of returns of thirty-six U.S. and European stock market indices during periods of uncertainty. Specifically, the authors studied the period of the Global Financial Crisis as well as the period of the COVID-19 pandemic. Their results showed that entropy decreased during the periods of turbulence, indicating that the regularity and predictability of stock market indices returns increased during these periods. In the field of Econophysics, the study of the complexity and multifractality of financial time series during the pandemic is a challenging topic. However, to the best of our knowledge, until now there has not been an in-depth comparative analysis of the effects of the pandemic on the complexity and fractal characteristics of the returns of two completely different currencies, such as BTC/USD and EUR/USD, that play a key role in the modern financial system.

In this article, we present a study of the temporal evolution of the multifractality and complexity of BTC/USD and EUR/USD returns for the period before and after the WHO announcement that declared COVID-19 a global pandemic (i.e., 11 March 2020). We chose to analyze and compare the effects of the pandemic on the two most representative currencies from the cryptocurrency and forex markets, respectively. Although these two markets are completely different from each other, they play a significant role in the modern financial system. More specifically, we applied the A-MF-DFA to investigate the temporal evolution of the asymmetric multifractal spectrum parameters $\left(\alpha_{0},\;\Delta \alpha ,\;A\right)$ before and after the outbreak of the pandemic. Although there are numerous studies that have followed a similar approach for the study of financial time series (e.g., [37,38,39]), as far as we are able to know, this is the first time that the temporal evolution of the specific parameters has been applied to BTC/USD and EUR/USD returns to study the period before and during COVID-19. At this point, it is important to mention that the analysis of the multifractal properties of financial time series has a wide contribution to the field of finance. For example, multifractality can be used to obtain better forecasts of tail risk as demonstrated by Batten et al. [40]. In addition, we examined the temporal evolution of four popular complexity measures. Although approximate and sample entropies are quite common for financial time series analysis [31,35,41], we chose to use Fuzzy entropy as it is considered as an upgraded alternative of approximate and sample entropy for evaluating the complexity, specifically for short time series contaminated by noise [42]. In combination, we chose to use the Shannon entropy as the standard information measure and Tsallis entropy as its non-extensive generalization, very closely related to multifractality. Additionally, we used another complexity measure, Fisher information. In financial data analysis, the application of Fisher information is very widespread for the construction of the Shannon–Fisher causality plane [34,43]. In the present article, we chose to investigate the temporal evolution of Fisher information as we believe that it can reveal useful elements for the evolution of the complexity of the dynamical system, providing a “mirror image” of the evolution of entropies, but also presenting the key difference of its so-called “locality” property (see Section 2.4). Our study attempts to provide a complete picture of the pandemic’s impact in terms of the dynamical change of the complexity and the fractal characteristics of the two currencies. Additionally, our results provide useful conclusions about the behavior of two very different currencies during uncertainty periods. At the same time, interesting conclusions are drawn about the impact of WHO announcements and the reaction of investors to external events such as the pandemic. Our findings can help both investors and risk managers, as well as policymakers, to formulate a comprehensive response to the occurrence of such external events.

## 2. Materials and Methods

This section briefly presents the asymmetric multifractal detrended fluctuation analysis approach (Section 2.1), as well as key concepts of multifractal spectrum parameters (Section 2.1.1). Additionally, we present key notions and formulae related to Fuzzy entropy (Section 2.2), Tsallis entropy (Section 2.3), and Fisher information measure (Section 2.4).

### 2.1. Asymmetric Multifractal Detrended Fluctuation Analysis (A-MF-DFA)

The A-MF-DFA extends the MF-DFA method by considering positive and negative market trends [44,45]. First, the profile time series of each return time series $\left\{x_{j\;}:j=1,\ldots ,N\right\}$ are calculated as $X\left(t\right)=\sum_{j=1}^{t}\left(x_{j}-\bar{x}\right)$ for t=1,…, N, where $\bar{x}$ is the average of the entire return time series. Then, the profile time series and the return time series are both divided into $N_{n}=\lfloor N/n\rfloor$ non-overlapping segments of length n. In case N is not a multiple of n, we repeat the division initially from the other end of the time series to take into account all the available data, making a total of $2N_{n}$ segments for both the profile and the return time series.

Next, the local trend of the profile series ${\tilde{X}}_{v}\left(i\right),\;i=1,\ldots ,n$ is calculated for each segment $v=1,\ldots ,\;2N_{n}$, by fitting a least-square polynomial of degree 2 in order to detrend the corresponding profile $X_{v}\left(i\right),\;i=1,\ldots ,\;n.$ For the return time series, the local linear trend for each segment is also calculated to determine whether the return time series show an uptrend or downtrend. The different trends depend on the sign of each local slope $b_{n,v}\neq 0$, where $b_{n,v}$ represents the coefficient of the linear trend for segment v at scale n [27]. If $b_{n,v}>0$ ($b_{n,v}<0$), the return time series have an upward (downward) trend within the vth segment.

Then, we define the residual variance as follows:

(1) $$F^{2}\left(n,\;v\right)=\frac{1}{n}\overset{n}{\underset{i=1}{\sum }}{\left(X_{v}\left(i\right)-{\tilde{X}}_{v}\left(i\right)\right)}^{2}.$$

By taking the average over corresponding segments, we can obtain the asymmetric qth order average fluctuation functions, which are then calculated by taking the average over the corresponding segments:

(2) $$F_{q}^{+}\left(n\right)={\left\{\frac{1}{M^{+}}\;\overset{2N_{n}}{\underset{v=1}{\sum }}\frac{1+\mathrm{sgn}(b_{n,\;v})}{2}{\left[F^{2}\left(n,\;v\right)\right]}^{\frac{q}{2}}\right\}}^{\frac{1}{q}},$$

(3) $$F_{q}^{-}\left(n\right)={\left\{\frac{1}{M^{-}}\;\overset{2N_{n}}{\underset{v=1}{\sum }}\frac{1-\mathrm{sgn}(b_{n,\;v})}{2}{\left[F^{2}\left(n,\;v\right)\right]}^{\frac{q}{2}}\right\}}^{\frac{1}{q}},$$

where $M^{+}=\sum_{v=1}^{2N_{n}}\left(1+\mathrm{sgn}(b_{n,\;v}\right))/2$ and $M^{-}=\sum_{v=1}^{2N_{n}}\left(1-\mathrm{sgn}(b_{n,\;v}\right))/2$ are the number of total segments with directional trends. Note that for all $v=1,\;\ldots ,2N_{n}$, $\;M^{+}+M^{-}=2N_{n}$ holds. Therefore, the qth order average fluctuation functions for the overall trend is written as:

(4) $$F_{q}\left(n\right)={\left\{\frac{1}{2N_{n}}\overset{2N_{n}}{\underset{v=1}{\sum }}{\left[F^{2}\left(n,\;v\right)\right]}^{\frac{q}{2}}\right\}}^{1/q}.$$

The calculation is repeated to find the fluctuation function for all box sizes n. If long-range power-law correlations are present, the function will increase with n as a power-law $F_{q}\left(n\right)∼n^{h\left(q\right)}$. The scaling exponent $h\left(q\right)$, namely, the generalized Hurst exponent, is calculated by estimating the slope of the linear regression of $\log\left(F_{q}\left(n\right)\right)$ versus $\log\left(n\right)$. The asymmetric generalized exponents $h^{+}\left(q\right)$ and $h^{-}\left(q\right)\;$ are calculated in a similar way from the relationship $F_{q}^{+}\left(n\right)∼n^{h^{+}\left(q\right)}$ and $F_{q}^{-}\left(n\right)∼n^{h^{-}\left(q\right)}.$ In this study, we consider n ranging from 8 to N/4 for the log-log linear regression to estimate the asymmetric generalized Hurst exponents.

#### 2.1.1. Asymmetric Multifractal Spectrum Parameters

The multifractal characteristics of time series can be described not only by the generalized Hurst exponent $H\left(q\right)$ but also by the multifractal scaling exponent $\tau \left(q\right)$, and their relationship can be expressed as $\tau \left(q\right)=qH\left(q\right)-1$. In the case that $\tau \left(q\right)$ and q are linearly related, the analyzed time series is monofractal. In the case that $\tau \left(q\right)$ and q have a nonlinear relationship, the analyzed time series is multifractal. Additionally, it is significant to note that the stronger their nonlinear relationship is, the stronger the multifractal characteristics are [46].

Moreover, using the multifractal (singularity) spectrum $f\left(\alpha \right)$ can also describe multifractional characteristics of time series. The multifractal spectrum is obtained by applying the first-order Legendre transform [39,46]:

(5) $$\alpha =d\tau \left(q\right)/dq,$$

(6) $$f\left(\alpha \right)=q\alpha -\tau \left(q\right),$$

where α is the singularity strength (also known as the Hölder exponent) that characterizes singularities in the time series. The interpretation of α is as follows: If α=1, then the distribution of the time series data is uniform. If α<1, then the singularity degree is larger. On the other hand, if α>1, then the singularity degree is smaller. The multifractal spectrum $f\left(\alpha \right)$ denotes the singularity content [46,47].

To analyze and make a solid understanding of the multifractal characteristics of a time series, a set of the asymmetric multifractal spectrum parameters $\left(\alpha_{0},\;\Delta \alpha ,\;A\right)$ has been suggested. More specifically, the maximum of the multifractal spectrum $f\left(\alpha \right)$ is used to detect the correlation behavior in terms of persistence and anti-persistence. The spectrum $\alpha_{0}$ gives the maximum $f\left(\alpha \right)$, i.e., $f\left(\alpha_{0}\right)=1$. At this spectrum, the measure provides information about the central tendency of the multifractal spectrum. If $\alpha_{0}<0.5$, then the correlations in the time series exhibit anti-persistent behavior (i.e., an increase is very likely to be followed by a decrease), if $\alpha_{0}>0.5$, then the correlations in the time series exhibit persistent behavior (i.e., an increase is very likely to be followed by an increase, and a decrease is very likely to be followed by a decrease), whereas if $\alpha_{0}=0.5$, then the time series displays characteristics of a standard non-correlated sequence [39,47,48]. By looking into the spectrum width, one can quantitatively detect the time series multifractality. Specifically, the width of the spectrum is estimated by the equation $\Delta \alpha =\alpha_{max}-\alpha_{min}$, and it reflects the degree of multifractality of the time series. The larger values of Δα are, the stronger the degree is and the more severe the fluctuations in the time series are. On the contrary, the smaller the values of Δα, the more the time series is close to a monofractal behavior, indicating less significant fluctuations in the time series. The spectrum width should be equal to zero for a completely monofractal time series [39,49,50]. The dominance of small or large fluctuations is also an interesting characteristic of time series. This information can be extracted from the skew asymmetry of the multifractal spectrum, which is defined by the equation [51] $A=\frac{L-R}{R+L}=\frac{-\Delta S}{W}$, where $R=\alpha_{max}-\alpha_{0}$, $L=\alpha_{0}-\alpha_{min}$, ΔS=R−L, and $W=R+L=\Delta \alpha =\alpha_{max}-\alpha_{min}$. If $A>0\;\left(L>R\right),$ the spectrum is left-skewed, which means that the scaling behavior of large fluctuations dominates the multifractal behavior. On the contrary, if $A<0\;\left(L<R\right),$ then the spectrum is right-skewed, where the scaling behavior of small fluctuations dominates. The case of A=0 indicates that the shape of multifractal spectra is symmetric [46,51].

Another multifractal spectrum asymmetry metric is the so-called truncation, defined as $\Delta f\left(a\right)=f\left(\alpha_{min}\right)-f\left(\alpha_{max}\right)$ [49,52]. If $\Delta f\left(a\right)<0$, the multifractal spectrum is right-truncated, i.e., it has a long left tail, indicating that the multifractal structure in the time series is insensitive to the local fluctuations with small magnitudes. In other words, the time series is less multifractal, closer to monofractal, for the small fluctuations than for the large fluctuations. If $\Delta f\left(a\right)>0$, the multifractal spectrum is left-truncated, i.e., it has a long right tail, indicating that the multifractal structure is then insensitive to the local fluctuations with large magnitudes. It has to be noted that, very often, truncation and skew asymmetries are directly related so that a left-skewed spectrum is also right-truncated, and a right-skewed is left-truncated. The absolute value of truncation, also known as “C-value”, $C-\mathrm{value}=\left|\Delta f\left(a\right)\right|=\left|f\left(\alpha_{min}\right)-f\left(\alpha_{max}\right)\right|$ [49,52], indicates the degree of the truncation asymmetry, which also provides interesting information as C-values are known to illustrate the systems’ underlying undulation or instability. The degree of undulation or instability becomes minimum when the C-value presents the smallest value (≈ 0) [49,52].

### 2.2. Fuzzy Entropy (FuzzyEn)

Expanding upon the concepts already established with approximate entropy (ApEn) and sample entropy (SampEn), Chen et al. [53,54] combined elements from Fuzzy sets and information theory to develop a fuzzy version of the SampEn. Fuzzy entropy (FuzzyEn) like its ancestors, ApEn and SampEn [54], is a “regularity statistic” that quantifies the (un)predictability of fluctuations in a time series. For the estimation of FuzzyEn, the similarity between vectors is defined based on fuzzy membership functions and the vectors’ shapes. The gradual and continuous boundaries of the fuzzy membership functions lead to a series of advantages, such as the continuity as well as the validity of FuzzyEn at small values, higher accuracy, stronger relative consistency, and even less dependence on the data length. FuzzyEn can be considered as an upgraded alternative of SampEn (and ApEn) for the evaluation of complexity, especially for short time series contaminated by noise [55].

Similar to SampEn, FuzzyEn excludes self-matches. Nevertheless, it applies a slightly different definition for the employed first N−m vectors of a length of m, by removing a baseline, ${\bar{s}}_{i}$:

(7) $${\bar{s}}_{i}=m^{-1}\overset{m-1}{\underset{j=0}{\sum }}s_{i+j},$$

i.e., for the FuzzyEn estimations, we use the first N−m of the vectors:

(8) $$X_{i}^{m}=\left\{s_{i},\;s_{i+1},\ldots ,s_{i+m-1}\right\}-{\bar{s}}_{i},\;\;i=1,\;2,\ldots ,\;N-m+1,$$

Then, the similarity degree, $D_{ij}^{m}$, between each pair of vectors, $X_{j}^{m}$ and $X_{i}^{m}$, being within a distance, r, from each other is defined by a fuzzy membership function:

(9) $$D_{ij}^{m}=\mu \left(d_{ij}^{m},r\right),$$

where $d_{ij}^{m}$ is, as in the case of ApEn and SampEn, the supremum norm difference between $X_{i}^{m}$ and $X_{j}^{m}$. For each vector, $X_{i}^{m}$, we estimate the average similarity degrees with respect to all other vectors, $X_{j}^{m},\;j=1,2,\ldots ,N-m+1$, and j≠i (i.e., excluding itself):

(10) $$\phi_{i}^{m}\left(r\right)={\left(N-m-1\right)}^{-1}\overset{N-m}{\underset{i=1,j\neq i}{\sum }}D_{ij}^{m}.$$

Then, we evaluate

(11) $$\varphi^{m}\left(r\right)={\left(N-m\right)}^{-1}\overset{N-m}{\underset{i=1}{\sum }}\phi_{i}^{m}\left(r\right),$$

and

(12) $$\varphi^{m+1}\left(r\right)={\left(N-m\right)}^{-1}\overset{N-m}{\underset{i=1}{\sum }}\phi_{i}^{m+1}\left(r\right).$$

The $FuzzyEn\left(m,r\right)$ is then defined as

(13) $$FuzzyEn\left(m,r\right)=\underset{N\to \infty }{\lim}\left[\ln\varphi^{m}\left(r\right)-\ln\varphi^{m+1}\left(r\right)\right],$$

which, for finite time series, can be calculated by the statistic

(14) $$FuzzyEn\left(m,r,N\right)=\ln\varphi^{m}\left(r\right)-\ln\varphi^{m+1}\left(r\right).$$

As mentioned above, FuzzyEn is a measure of estimation of the complexity. More specifically, lower FuzzyEn values demonstrate a larger chance that a set of data will be followed by similar data (regularity). Hence, lower values demonstrate larger regularity. Conversely, a greater value of FuzzyEn indicates a smaller chance of similar data being repeated (irregularity). Thus, greater values convey more randomness, disorder, and system complexity. Consequently, a low (high) value of FuzzyEn reflects a high (low) degree of regularity [42].

### 2.3. Tsallis Entropy

In a vast variety of systems that exhibit long-range interactions or long-term memory or being of a multifractal nature, they have been found to be better described by a generalized statistical-mechanical formalism proposed by Tsallis [56,57]. Tsallis, inspired by multifractal concepts, introduced an entropic expression characterized by an index, $q_{TS}$, which leads to non-extensive statistics [56,57]:

(15) $$S_{q_{TS}}=k\frac{1}{q_{TS}-1}\left(1-\overset{W}{\underset{i=1}{\sum }}p_{i}^{q_{TS}}\right),$$

where $q_{TS}$ is a real number, k is the Boltzmann’s constant from statistical thermodynamics, $p_{i}$ are probabilities associated with the microscopic configurations, and W is their total number. It is important to note that there is a remarkable conceptual similarity between Tsallis’ entropy definition and the notion of Rényi entropies.

The entropic index, $q_{TS}$, describes the deviation of Tsallis entropy from the standard Boltzmann–Gibbs entropy. Indeed, using $p_{i}^{\left(q_{TS}-1\right)}=e^{\left(q_{TS}-1\right)\ln\left(p_{i}\right)}\;\sim \;1+\left(q_{TS}-1\right)\ln\left(p_{i}\right)$ in the limit $q_{TS}\to 1$, we recover the Boltzmann–Gibbs entropy

(16) $$S_{1}=-k\overset{W}{\underset{i=1}{\sum }}p_{i}\ln\left(p_{i}\right),$$

as the thermodynamic analog of the information-theoretic Shannon entropy. From this point and for the rest of this article, we will refer to the entropy calculated by Equation (16) as the Shannon entropy.

For $q_{TS}\neq 1$, the entropic index, $q_{TS}$, characterizes the degree of non-extensivity reflected in the following pseudo-additivity rule:

(17) $$\frac{S_{q_{TS}}\left(A+B\right)}{k}=\frac{S_{q_{TS}}\left(A\right)}{k}+\frac{S_{q_{TS}}\left(B\right)}{k}+\left(q_{TS}-1\right)\frac{S_{q_{TS}}\left(A\right)}{k}\frac{S_{q_{TS}}\left(B\right)}{k},$$

where A and B are two subsystems. In case these subsystems have special probability correlations, extensivity does not hold for $q_{TS}=1\;\left(S_{1}\neq S_{1}\left(A\right)+S_{1}\left(B\right)\right)$, but may occur for $S_{q_{TS}}$, with a particular value of the index, $q_{TS}\neq 1$. Such systems are called non-extensive [56]. The cases $q_{TS}>1$ and $q_{TS}<1$ correspond to sub-additivity or super-additivity, respectively. As in the case of Rényi entropies, we may think of $q_{TS}$ as a bias parameter: $q_{TS}<1$ privileges rare events, while $q_{TS}>1$ highlights prominent events [58].

It is noted that the parameter, $q_{TS}$, itself is not a measure of the complexity of the system but measures the degree of the non-extensivity of the system. In turn, the temporal variations of the Tsallis entropy, $S_{q_{TS}}$, for some $q_{TS}$, quantify the dynamical changes of the complexity of the system. In particular, lower $S_{q_{TS}}$ values characterize the portions of the signal with lower complexity [55].

### 2.4. Fisher Information Μeasure (FIM)

In the last decades, Fisher information has been increasingly gaining the interest of scientists of different scientific fields. It was first introduced by Fisher [59] as a representation of the amount of information in the results of experimental measurements of an unknown parameter of a stochastic system, or simply the amount of information that can be extracted from a set of measurements (or the “quality” of the measurements) [60]. Fisher information is a useful method to study non-stationary and complex time series [61]. It is used as a measure of the level of disorder of a system, behaving inversely to entropy, i.e., when the disorder increases, the entropy increases, while the Fisher information decreases. Fisher information has been successfully applied to many different systems, revealing its ability in describing the complexity of them [62,63,64]. Additionally, its use has been suggested to identify reliable precursors of critical events [65,66,67]. Moreover, Fisher information presents the so-called “locality” property in contrast to the “globality” of entropy, referring to the sensitivity of Fisher information in changes in the shape of the probability distribution corresponding to the measured variable, not presented by entropy [68,69]. The Fisher information measure can be expressed as

(18) $$I_{x}=\sum_{n=1}^{N-1}\frac{{\left[p\left(x_{n+1}\right)-p\left(x_{n}\right)\right]}^{2}}{p\left(x_{n}\right)}.$$

The discrete probability distribution $p\left(x_{n}\right)$ corresponds to the specific values of the unknown underlying probability density function at the center values of the intervals $\left\{x_{n}\right\}$, which are not necessarily of equal length. The probability density function is usually approximated by a histogram, or by the kernel density estimator technique, employing different kernel functions such as the Gaussian kernel or Epanechnikov kernel [60].

## 3. Data and Results

The cryptocurrency market is a relatively new and emerging market, meaning that the trading mechanism is unique and makes it very different from traditional markets. More than 21,800 different cryptocurrencies are currently traded around the world with an estimated total market capitalization of over USD 843 billion (see, e.g., https://coinmarketcap.com/ (accessed on 7 December 2022)). On the other hand, the foreign exchange market is the largest financial market worldwide, with transactions amounting to trillions of US dollars daily [70]. In this article, we focused on the analysis of the two most representative currencies of these two markets, i.e., the BTC/USD and EUR/USD. Our analyses were applied to the daily logarithmic returns ($r_{t}=\ln p_{t}-\ln p_{t-1}$, where $p_{t}$ denotes the price at time t) of the BTC/USD and EUR/USD during the period from 1 May 2019 to 20 January 2021. In an announcement by the WHO on 11 March 2020, the outbreak of COVID-19 was declared a global pandemic. Therefore, we considered the period from 1 May 2019 to 11 March 2020 as the pre-announcement period, and the period from 12 March 2020 to 20 January 2021 as the post-announcement period. All financial time series were taken from Yahoo Finance (http://finance.yahoo.com/ (accessed on 7 December 2022)).

In our study, we investigated the temporal evolution of complexity and fractal characteristics by using overlapping sliding windows (with window length equal to 512 samples and slide step equal to 1 sample). First, we investigated the temporal evolution of the multifractal spectrum parameters $\left(\alpha_{0},\;\Delta \alpha ,\;A\right)$ before and after the outbreak of the pandemic. Then, for the same time period, we extended our analysis by examining the temporal evolution of Fuzzy entropy, Tsallis entropy, Shannon entropy, and Fisher information.

At this point, we should mention that for the calculation of Tsallis entropy we have chosen to use the value $q_{TS}=1.8$ for the non-extensive parameter, $q_{TS}$. On one hand, for financial time series $q_{TS}$ has been found to take values $q_{TS}\sim 1.6-1.8$ [3], which has been discussed within the framework of the similarities in scaling properties and universality related to observables of extreme events from different disciplines (e.g., financial crisis, earthquake, epileptic seizure, magnetic storm, solar flare) [3,55,60,61]. On the other hand, from the time series analysis point of view, the selection of the $q_{TS}$ value for the calculation of the temporal variation of Tsallis entropy practically only affects the “separation” between the lower and higher complexity parts of the analyzed time series (e.g., min to max entropy values ratio, in direct analogy to the signal to noise ratio), while for the herein analyzed time series it was found that any $q_{TS}$ value in the range ~1.6−1.8 leads to approximately the same results.

**Figure 1:**
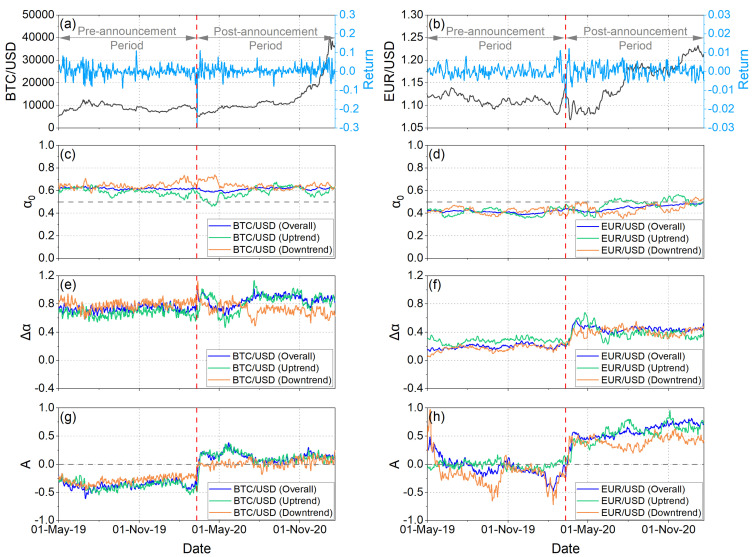
Comparative asymmetric multifractal analysis of BTC/USD (left panels) and EUR/USD (right panels) under different market trends. (**a**,**b**): Exchange rates and Returns. (**c**,**d**): Temporal evolution of $\alpha_{0}$ parameter. (**e**,**f**): Temporal evolution of width of the multifractal spectrum. (**g**,**h**): Temporal evolution of the asymmetry parameter A values. The red vertical dash line corresponds to the date of the WHO announcement in which COVID-19 was declared a global pandemic (i.e., 11 March 2020). The period from 1 May 2019 to 11 March 2020 corresponds to the pre-announcement period, while the period from 12 March 2020 to 20 January 2021 corresponds to the post-announcement period.

Figure 1c,d, depict the temporal evolution of $\alpha_{0}$ values under different market trends of the BTC/USD and EUR/USD returns, respectively. By analyzing the overall trend of the BTC/USD returns, it is observed that the values of the $\alpha_{0}$ fluctuate around 0.6 (Figure 1c). These results indicate that the returns time series is characterized by long-range correlations, both before and after the onset of COVID-19. By analyzing the downtrend markets of the BTC/USD returns, it is observed that the values of the $\alpha_{0}$ fluctuate over 0.6 both before and after the outbreak of the pandemic, indicating persistent behavior. In the uptrend markets of the BTC/USD returns, the values of $\alpha_{0}$ fluctuate between 0.5 and 0.65 for almost throughout the analysis period. An exception is a short period of time after the WHO announcement, where $\alpha_{0}$ values fell below 0.5.

Figure 1d depicts the temporal evolution of the $\alpha_{0}$ values of the EUR/USD returns under different market trends. In this case, we observe that for the overall trend the $\alpha_{0}$ values fluctuate around 0.4 during the whole pre-announcement period, while after the announcement they present a progressive increase approaching very close to $\alpha_{0}=0.5$ at the end of the considered analysis period. This suggests that the time series exhibit a “different” power-law correlation, such that large and small time series are more likely to alternate (anti-persistent behavior). It is worth mentioning that the downtrend $\alpha_{0}$ values remain at the anti-persistent side except for the very last part of the analyzed period, while the uptrend $\alpha_{0}$ values, although <0.5 for the whole pre-announcement period, present an alternating behavior after the WHO announcement, taking values $\alpha_{0}>0.5$ for two two-month-long periods.

Figure 1e,f illustrate the width of the multifractal spectrum under different market trends of the BTC/USD and EUR/USD returns, respectively. As already mentioned in Section 2.1.1, the width of the multifractal spectrum Δα is a measure of the degree of multifractality. If a time series presents a smaller width of the multifractal spectrum, this indicates that the time series has lower heterogeneity, i.e., lower fluctuations and lower market risk [58]. The results show that throughout the period analyzed, the width of the multifractal spectrum receives higher values for the BTC/USD returns compared to the EUR/USD returns. Therefore, it can be concluded that EUR/USD is relatively more stable than BTC/USD. In addition, we observe that after the outbreak of the pandemic, the width of the multifractal spectrum increased for both BTC/USD returns and the EUR/USD returns for the overall trend. This suggests that after the outbreak of the pandemic, both currencies reacted similarly in terms of multifractality when observed from an overall trend point of view. The degree of multifractality increased, and, therefore, the fluctuations became more intense and the market risk increased. However, in terms of asymmetric multifractality, this is not always the case. When focused on downside markets of BTC/USD, the degree of multifractality decreased after the outbreak. More interestingly, downtrend multifractality was higher than the uptrend multifractality during the period before COVID-19, but during the pandemic the uptrend multifractality became higher. These findings reveal that the incremental multifractality in BTC/USD is due to intense fluctuations and higher heterogeneity during price increases, but not during price declines. In EUR/USD, it appears that the downside markets play a more important role in increasing multifractality. Nevertheless, both market trends may have had some impact in the post-announcement period increase in multifractality. It is important to note that the increase in multifractality in BTC/USD returns during COVID-19 is consistent with the existing literature as other studies have reached the same conclusion (e.g., [26,27]).

Figure 1g,h depict the temporal evolution of the asymmetry parameter A values under different market trends of the BTC/USD and EUR/USD returns, respectively. In the time period before the onset of the pandemic, the asymmetry parameter A of BTC/USD returns appears to have been consistently below 0, indicating relative dominance of the small fluctuations. Immediately after the date of the WHO announcement, there was a sharp change in the values of A in all market trends. Specifically, for both overall trend and uptrend markets, the values of A of the BTC/USD returns remain above 0 for the entire period after the outbreak of the pandemic. This sharp change shows a transition from a period where small fluctuations predominate (before the pandemic) to a period where large fluctuations predominate (during the pandemic). In the downtrend markets, the values of parameter A are almost at 0 for the entire period after the outbreak of the pandemic, indicating that the spectrum became practically symmetrical (Figure 1g). On the other hand, the values of the asymmetry parameter A of the EUR/USD returns for the uptrend markets are almost equal to 0 for the whole period before the outbreak of the pandemic. This fact indicates that the spectrum is practically symmetrical. On the contrary, analyzing the overall and downward trends of the market, we observe that the values of the asymmetry parameter A are below 0, almost for the entire period before the outbreak of the pandemic. Therefore, it is concluded that in the overall and downward trends of the markets, they are dominated by the small fluctuations in EUR/USD returns before the outbreak of the pandemic. Immediately after the announcement date, the values of the asymmetry parameter A of the EUR/USD returns exceeded 0 in all market trends. This result shows that EUR/USD returns after the outbreak of the pandemic are dominated by large fluctuations (Figure 1h).

**Figure 2:**
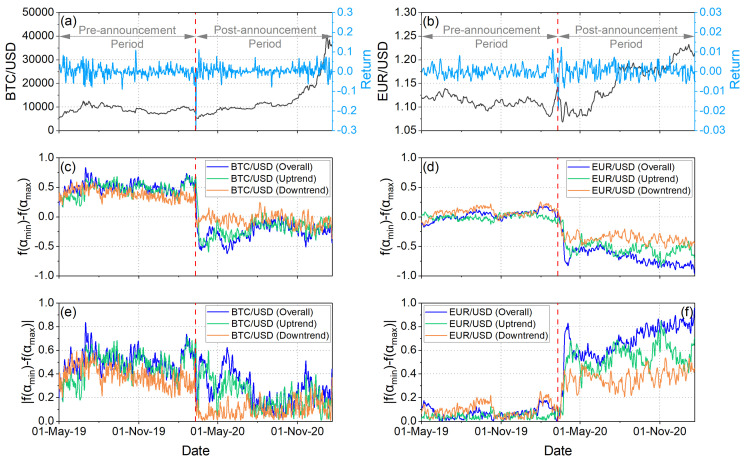
Comparative asymmetric multifractal analysis of BTC/USD (left panels) and EUR/USD (right panels) under different market trends. (**a**,**b**): Exchange rates and Returns. (**c**,**d**): Temporal evolution of truncation $\Delta f\left(a\right)=f\left(\alpha_{min}\right)-f\left(\alpha_{max}\right)$. (**e**,**f**): Temporal evolution of the degree of truncation asymmetry, known as $C-\mathrm{value}=\left|\Delta f\left(a\right)\right|=\left|f\left(\alpha_{min}\right)-f\left(\alpha_{max}\right)\right|$. The red vertical dash line corresponds to the date of the WHO announcement in which COVID-19 was declared a global pandemic (i.e., 11 March 2020). The period from 1 May 2019 to 11 March 2020 corresponds to the pre-announcement period, while the period from 12 March 2020 to 20 January 2021 corresponds to the post-announcement period.

Figure 2c,d indicate that the effect of the announcement was, for all cases (for both BTC/USD and EUR/USD returns and for all three considered market trends), a sharp change towards right-truncation, which means that after the WHO announcement the multifractal structure in the time series became more insensitive to the local fluctuations with small magnitudes. On the other hand, Figure 2e,f show that the behavior of BTC/USD and EUR/USD returns was different concerning the degree of truncation asymmetry, indicated by the so-called C−value. Specifically, EUR/USD returns present C−values quite close to 0 before the WHO announcement, which means that the underlying system then presented the lowest possible undulation or instability. After the WHO announcement, the picture changed and for all market trends an increase in the undulation or instability of the underlying system is observed. In contrast, BTC/USD returns present a general trend (although with notable fluctuations for the overall and uptrend markets) towards a decrease in the C−values after the WHO announcement, which means that a trend for the decrease in the undulation or instability of the underlying system is observed. It is noted that the downtrend market after the WHO announcement presents C−values closer to 0, as compared with the uptrend and overall markets, indicating lower undulation or instability.

**Figure 3:**
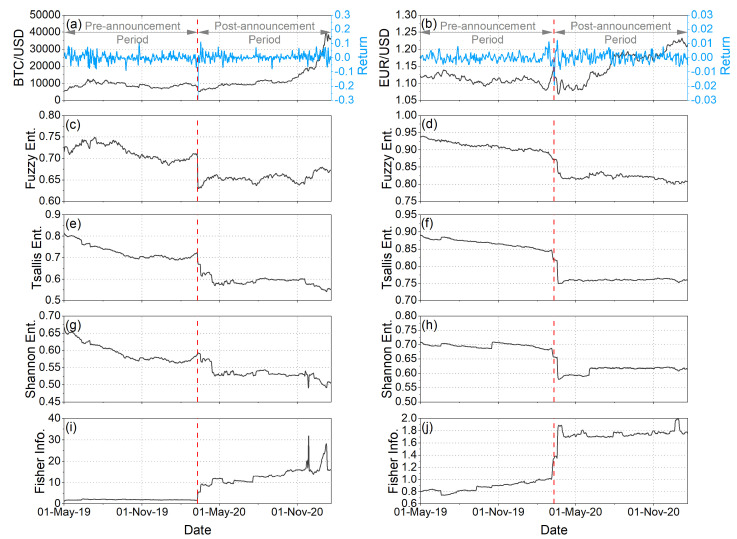
Comparative analysis of BTC/USD (left panels) and EUR/USD (right panels). (**a**,**b**): Exchange rates and Returns. (**c**,**d**): Temporal evolution of Fuzzy entropy. (**e**,**f**): Temporal evolution of Tsallis entropy. (**g**,**h**): Temporal evolution of Shannon entropy. (**i**,**j**): Temporal evolution of Fisher information. The red vertical dash line corresponds to the date of the WHO announcement in which COVID-19 was declared a global pandemic (i.e., 11 March 2020). The period from 1 May 2019 to 11 March 2020 corresponds to the pre-announcement period, while the period from 12 March 2020 to 20 January 2021 corresponds to the post-announcement period.

Moreover, we analyzed the temporal evolution of some complexity measures. Figure 3c,d illustrate the temporal evolution of the Fuzzy entropy of the BTC/USD and EUR/USD returns, respectively. As it has already been mentioned in Section 2.2., smaller values of Fuzzy entropy indicate a greater chance that a set of data will be followed by similar data (regularity). Conversely, larger values of Fuzzy entropy point to a lower chance of similar data being repeated (irregularity). As we observe in Figure 3c,d, the values of Fuzzy entropy dropped sharply in both BTC/USD and EUR/USD returns immediately after the WHO announcement. This fact indicates that in the pre-announcement period, both BTC/USD and EUR/USD returns were characterized by a higher degree of disorder and randomness, i.e., by higher complexity. In contrast, in the period during the pandemic, the values of Fuzzy entropy decreased, suggesting that the returns were characterized by a higher degree of order and lower complexity. Therefore, it is concluded that the pandemic led investors to behave in an “organized” (similar) way that thereby reduced the complexity of the two markets.

Corresponding results are obtained by also studying two quite popular complexity measures, i.e., the Shannon entropy (Figure 3g,h) and Tsallis entropy (Figure 3e,f). More specifically, the time variations of the Shannon entropy as well as the Tsallis entropy (for a given $q_{TS}$) quantify the dynamical changes of the information content and the complexity of the system. Smaller values characterize time series with lower complexity and randomness, as well as higher information content and order. Conversely, larger values characterize time series with higher complexity, disorder and randomness, as well as lower information content. As we observe in Figure 3e–h, during COVID-19, the values of Tsallis and Shannon entropies were reduced in both BTC/USD and EUR/USD returns, indicating that the complexity of the two markets was reduced and the information content was increased. It is important to note that all the entropy measures we applied quickly adapted to market conditions, showing a sharp decrease immediately after the WHO announcement, with Shannon entropy being the exception in the case of BTC/USD. Additionally, it is of particular interest that the entropy values remained at low levels throughout the pandemic period we analyzed, showing that the effects of the pandemic were not short-term. Additionally, concerning the study of Lahmiri and Bekiros [32], although not the main finding of their analyses, it is nevertheless important to note that their results showed a decrease in Rényi entropy (and consequently a decrease in randomness) for the BTC/USD market during the pandemic compared to before.

In addition, we applied one more complexity measure, the Fisher information. Fisher information is a useful method to study non-stationary and complex time series. Fisher information is used as a measure of the degree of order of a system, behaving inversely to entropy, i.e., when the order increases, the entropy decreases, while the Fisher information increases. Moreover, unlike entropy, it is sensitive to changes in the shape of the probability distribution corresponding to the measured variable. Figure 3i,j illustrate the temporal evolution of the Fisher information of the BTC/USD and EUR/USD returns, respectively. We observe that immediately after the WHO announcement, the values of Fisher information increased in both BTC/USD and EUR/USD returns, indicating an increase in the order of the two markets.

At this point, it has to be mentioned that the observed decrease in randomness after the WHO announcement, indicated by all the applied complexity measures, is fully compatible with the corresponding increase of multifractality. Specifically, the more random a time series is, the more unifractal its scaling is, which means that a more multifractal time series can be considered as being farther away from “randomness” [71].

From the interpretation of our results in financial terms, useful conclusions are revealed. More specifically, in analyzing the values of $\alpha_{0}$ for overall trend, as we have already mentioned, we observe that the BTC/USD returns show persistent behavior, while the EUR/USD returns exhibit anti-persistent behavior almost throughout the time period we studied them (Figure 1c,d). A persistent or anti-persistent market return series is characterized by a long memory effect. Therefore, what happens today, theoretically, will impact the future in a nonlinear fashion. For example, if a persistent market return change has been up (down) in the last period, then the changes will continue to be positive (negative) in the next period. On the other hand, anti-persistent markets are “mean-reverting.” If the market return was up (down) in the previous period, it is more likely to be down (up) in the next period [72]. The long-memory characteristic in asset return is a fascinating topic for investors, risk managers, and scholars since appropriate return modeling is crucial for asset allocation and risk control. For example, existence of long memory in asset returns indicates that historical returns changes could be predictors of future returns changes [73]. Then, analyzing the Δα and A parameters, we observe that in the post-announcement period, mainly in the case of the EUR/USD, the degree of multifractal returns increased, and, therefore, fluctuations became more intense and market risk increased (Figure 1e,f). At the same time, we observe that in the post-announcement period, returns were dominated by large fluctuations (Figure 1g,h). Therefore, it is concluded that in the post-announcement period, EUR/USD returns experienced intense and large fluctuations. Similar behavior is observed for the overall trend and uptrend markets of the BTC/USD returns. Regarding the downtrend markets of the BTC/USD returns, it appears that during the pandemic period there were less intense fluctuations compared to the pre-pandemic period without small or large fluctuations in returns dominating. The analysis of the truncation asymmetry degree (Figure 2e,f), moreover, revealed that the WHO announcement had different impacts on BTC/USD and EUR/USD returns concerning the undulation or instability of the underlying system. For EUR/USD returns, the post-announcement period was characterized by an increase in the undulation or instability of the underlying system, whereas for BTC/USD returns, the opposite behavior was generally observed. Analyzing the complexity measures (Fuzzy entropy, Tsallis entropy, Shannon entropy, and Fisher information) (Figure 3c–j), we observe a sharp decline in complexity (i.e., increase in the order and information content, and decrease in randomness) in the returns of both BTC/USD and EUR/USD in the post-announcement period. This fact, in financial terms, suggests that the pandemic led investors to behave in an “organized” (similar) way that thereby reduced the complexity of the two markets. In other words, after the outbreak of the pandemic, it seems that investors behaved like a herd. Therefore, it is concluded that although the fluctuations were larger and more intense after the outbreak of the pandemic, this was not carried out in a random way as investors seem to have behaved in an “organized” way; however, this behavior for BTC/USD returns was generally followed by a decrease in undulation or instability of the underlying system, while the opposite happened for EUR/USD returns.

Additionally, it is worth noting that the majority of the measures that we studied showed a strong change for both BTC/USD and EUR/USD returns immediately after the WHO announcement (11 March 2020), in which COVID-19 was mentioned for the first time as a pandemic. This fact indicates that the behavior of the system changed immediately after the WHO’s announcement, although the discussions about COVID-19 being a public health emergency of international concern had begun weeks before. Therefore, although many researchers accept the date of 2 January 2020 as the beginning of the COVID-19 pandemic crisis (e.g., [74,75,76]), we consider the most suitable start date of the pandemic to be 11 March 2020.

## 4. Conclusions

The detection of dynamical complexity in time series originated from various complex systems, including the disciplines of physics, finance, and medicine, and is one of the foremost problems in science. The measurement of complexity includes nonlinear statistics methods to extract hidden patterns as well as exploring multifractality, randomness, and information flows. Hence, complexity provides important information regarding the order or disorder states of a system under scrutiny. In the field of finance, the detection of the dissimilarity of complexity between order and disorder states (e.g., before and after the occurrence of extreme events) could shed light on the mechanisms associated with investor reaction to these events.

In this article, we studied the temporal evolution of the multifractality and complexity of BTC/USD and EUR/USD returns for the period before and after the WHO announcement that declared COVID-19 a global pandemic. In our study, we first examined the asymmetric multifractality through the analysis of the multifractal spectrum parameters as obtained by the A-MF-DFA method. Then, we extended our analysis by applying Fuzzy, Tsallis, and Shannon entropies as well as the Fisher information measure. Our results can be summarized as follows: (i) For the entire time period that we studied (i.e., before and during the pandemic), the behavior of BTC/USD returns was persistent in all trends of the market. On one hand, in the period before the outbreak of the pandemic, the behavior of EUR/USD returns was anti-persistent in all trends of the market. On the other hand, in almost the entire period after the outbreak of the pandemic, the returns of the EUR/USD exhibited anti-persistent behavior in the overall trend and downtrend markets, while the uptrend market presented an alternating behavior, including short periods of persistent dynamics. (ii) Throughout the period analyzed, the width of the multifractal spectrum received higher values for the BTC/USD returns compared to the EUR/USD returns. This implies that the multifractality of the BTC/USD returns was higher than the multifractality of the EUR/USD returns. In addition, after the outbreak of the pandemic, in the overall trend and uptrend markets, the width of the multifractal spectrum increased for both BTC/USD returns and EUR/USD returns. In the case of BTC/USD, the downtrend multifractality was higher in the pre-announcement period. In EUR/USD, it appears that the downtrend markets played an important role in increasing multifractality. Nevertheless, both market trends may have had some impact on the post-announcement period increase in multifractality. (iii) In the pre-announcement period, small fluctuations in BTC/USD returns for all market trends dominated. In contrast, in the post-announcement period, large fluctuations in BTC/USD returns for overall trend and uptrend markets dominated, while in downtrend markets the spectrum became practically symmetrical. On the other hand, although in the uptrend markets the spectrum of EUR/USD returns was almost symmetrical, the returns in the overall trend and downtrend markets were dominated by small fluctuations for almost the entire pre-announcement period. During the pandemic period, the returns of the EUR/USD were dominated by large fluctuations in all market trends. (iv) For both BTC/USD and EUR/USD returns and all market trends, a sharp change towards becoming more insensitive to the local fluctuations with small magnitudes was observed after the WHO announcement. Nevertheless, the WHO announcement had different impacts on BTC/USD and EUR/USD returns concerning the undulation or instability of the underlying system. For EUR/USD returns, the post-announcement period was characterized by an increase in the undulation or instability of the underlying system, whereas for BTC/USD returns, the opposite behavior was generally observed. (v) Fuzzy entropy, non-extended Tsallis entropy, Shannon entropy, and Fisher information showed a sharp decrease in the degree of complexity immediately after the WHO announcement for both BTC/USD and EUR/USD. This fact shows that in the post-announcement period, the order and the information content of the systems increased, i.e., the randomness and complexity in the returns of the two currencies decreased. Therefore, in financial terms, we conclude that investors seem to have behaved in an “organized” way, as a herd. In addition, our analyses show that the date of the WHO announcement (11 March 2020) could be considered as the most appropriate date for the start of the pandemic. This element could be useful in future research studies.

The main finding that is revealed from our study is that immediately after the WHO announcement, the returns of both BTC/USD and EUR/USD presented a decrease in complexity and corresponding increase in multifractality, both indicating that they became less random compared to the pre-announcement period. Hence, it seems that although they are two such different currencies, which both play a key role in the modern financial system, they reacted in a similar way in response to the pandemic.

## Data Availability

All financial time series used in this article are publicly available from Yahoo Finance (http://finance.yahoo.com/, accessed on 7 December 2022).
